# Evaluation of the Feasibility of Transfusing Leukocyte Depletion Filter–Processed Intraoperative Cell Salvage Blood in Metastatic Spine Tumor Surgery: Protocol for a Non–Randomized Study

**DOI:** 10.2196/54609

**Published:** 2025-01-17

**Authors:** Naresh Kumar, Si Jian Hui, Renick Lee, Sahil Athia, Joel Yong Hao Tan, Jonathan Jiong Hao Tan

**Affiliations:** 1 Department of Orthopaedic Surgery, National University Hospital, National University Health System Singapore Singapore

**Keywords:** blood transfusion, autologous blood transfusion, operative blood salvage, leukocyte reduction filtration, intraoperative blood cell salvage, extramedullary spinal cord compression, metastases, tumors, leukocytes

## Abstract

**Background:**

Metastatic spine tumor surgery (MSTS) is often complex and extensive leading to significant blood loss. Allogeneic blood transfusion (ABT) is the mainstay of blood replenishment but with immune-mediated postoperative complications. Alternative blood management techniques (salvaged blood transfusion [SBT]) allow us to overcome such complications. Despite widespread use of intraoperative cell salvage (IOCS) in oncological and nononcological surgical procedures, surgeons remain reluctant to use IOCS in MSTS.

**Objective:**

This study aims to analyze safety of IOCS-leukocyte depletion filter (LDF)–processed blood transfusion for patients undergoing MSTS by assessing clinical outcomes—disease progression: tumor progression and overall survival. This study evaluates whether reinfusion of IOCS-LDF–processed blood reduces ABT rates in patients undergoing MSTS by sorting patients undergoing MSTS who require ABT into patients who consent to receive or not receive SBT.

**Methods:**

We aim to recruit a minimum of 90 patients—30 patients for SBT, 30 patients for ABT, and 30 patients with no blood transfusion. SBT and ABT form the 2 experimental arms, whereas no blood transfusion forms the control cohort. Available patient data will be reviewed to determine tumor burden secondary to metastasis and postoperative survival and disease progression, improvement in pain, and neurological and ambulatory status. Data collected will be studied postoperatively at 3, 6, 12, 24, 36, and 48 months or until demise, whichever occurs first. Outcomes of the experimental groups will be compared with those of the control group. Outcomes will be analyzed using 1-way ANOVA and Fisher exact test. The Kaplan-Meier curve and a log-rank test will be used to study overall survival. A multivariate and competing risk analysis will be used to study the association between blood transfusion type and tumor progression. All statistical analyses will be done using Stata Special Edition 14.0 (StataCorp LP).

**Results:**

This is the largest clinical study on use of IOCS in MSTS from various primary malignancies to date. It will provide significant clinical evidence regarding the safety and applicability of IOCS in MSTS. It will help reduce use of ABT, improving overall blood management of patients undergoing MSTS. A limitation of this study is that not all patients undergoing MSTS will survive for the follow-up period (4 years), theoretically leading to underreporting of disease progression. Study commenced in 2016 and patient recruitment continued till 2019. As of September 2019, we have collected operative data on 140 patients. However, the 2-year outcomes of about 40.0% (56/140) of patients are in the process of collection. The study is aimed to be published in the years 2023-2024.

**Conclusions:**

Results will be disseminated via peer-reviewed publications, paving the way for future studies.

**International Registered Report Identifier (IRRID):**

DERR1-10.2196/54609

## Introduction

### Background

The skeletal system is the third most common site of metastases, and cadaveric studies show that spinal metastases can be found in 30% to 90% of patients who die of cancer [1,2]. Surgery for metastatic spine disease (MSD) is complex, often requiring wide resection and extensive reconstruction leading to significant blood loss [1,2]. Emergency surgical treatment is often indicated for spinal cord compression with actual or impending paralysis or for spinal instability with severe pain that reduces the quality of life and mobility [3].

Typical blood loss in a patient undergoing tumor decompression and instrumentation in the thoracic and lumbar spine is about 1500 mL, and this requires an average of 3 units of packed red blood cells [4,5]. This blood loss is currently replenished by allogeneic blood transfusion (ABT) across the world, placing a significant burden on the already limited blood bank resources [6,7]. On the contrary, there is increasing evidence of the deleterious effects of ABT. Many studies have shown an increased incidence of postoperative infections [8] and promotion of tumor growth [8,9], which is thought to occur secondary to immunosuppression and other transfusion reactions.

Despite evidence describing multiple postoperative complications related to ABT [10,11], it remains to be the mainstay of blood replenishment in patients with heavy intraoperative blood loss [12]. These are immune-mediated complications and commonly affect the lungs (eg, transfusion-related acute lung injury) [13,14], wound healing [15-17], and survival duration [11,16]. This has prompted efforts to decrease reliance on ABT and to increase the utilization of alternatives, such as autologous predonation or intraoperative cell salvage (IOCS). Autologous predonation may not always be possible in metastatic spine tumor surgery (MSTS) because of suboptimal patient status, medical comorbidities, and in cases where emergency surgery is indicated.

Patient blood management is an evidence-based, patient-tailored approach aimed at reducing the need for ABT and its associated risks [18]. Patient blood management has both preoperative and perioperative components. Preoperative techniques include patient optimization via cessation of antiplatelet and anticoagulant medications [19,20] and anemia management [18]. Perioperative management comprises achieving surgical hemostasis, reinfusion of intraoperatively salvaged blood, the use of erythropoietic agents, and hemostatic drugs such as tranexamic acid. Randomized [21,22] and nonrandomized [23] studies on the use of IOCS in nononcological surgical procedures indicate that salvaged blood transfusion (SBT) significantly reduces the need for ABT [21-23]. Despite extensive use of SBT in orthopedic, trauma, and cardiac surgical procedures [24], the concern of reinfusion of tumor cells leading to disease progression persists [25-27]. The initial lack of literary evidence on the safety of SBT in oncological surgical procedures has made oncological surgeons reluctant to use SBT in MSTS [25-27].

This reluctance dates to an American Medical Council report from 1986, which stated that SBT was not suitable for use in tumor surgery [28]. This was in turn based on a case report from 1975 where tumor cells were found in salvaged blood [29]. There were concerns that tumor manipulation and resection would result in the spillage of tumor cells into the surgical field [30], which would lead to further metastasis if reinfused via SBT. Recent evidence indicates that circulating tumor cells (CTCs), which are shed by the primary tumor [31], are the most likely cause of tumor metastasis in oncological patients. CTCs have been shown to be eliminated by the reticular endothelial system [32], once they fail to metastasize (unable to complete the process of metastasis). Other CTCs may undergo cellular apoptosis after being retained in the capillary bed or the bone marrow [32]. These host defense mechanisms can prevent metastasis by reducing the metastatic ability of the vast majority of CTCs [33]. Consequently, one can ask, “Can SBT with a limited load of damaged malignant tumor cells cause tumor metastasis and disease progression?”

Investigation on the use of salvaged blood in MSTS started with a systematic review that we published [34]. It was envisaged that there is a place for salvaged blood in MSTS, provided the safety of IOCS in MSTS is established by the following steps. Our first step was to establish *basic cellular evidence* that there are no viable tumor cells in the salvaged blood. Second, we aimed to *quantify the number of tumor cells in the salvaged blood*, if any, and to demonstrate that the CTCs in the patient’s own blood are far more than those present in the salvaged blood. Our proposed *final phase* of study was to provide *clinical evidence* that SBT is safe for use in oncological surgical procedures, without increasing the risk of disease progression or tumor recurrence or resulting in poorer prognosis [32].

Our working hypothesis for the preclinical phase of this study was that the blood salvaged from patients undergoing MSTS does not contain viable tumor cells, and even if it did, viable tumor cells in the salvaged blood would be significantly lower than the number of CTCs present in the patient’s own blood at any given point in time. Therefore, there should be no increase in the risk of disease progression, in terms of further tumor dissemination, decreased survival, or increased tumor recurrence.

To test this hypothesis, we first conducted a study that analyzed the morphology and structural integrity of the tumor cells present in the patient’s circulation, operative field, and pre- and postfiltration samples of the salvaged blood [24,35]. Using the cell block technique, salvaged blood in the pre- and postfiltration samples was shown to mostly comprise cytoplasmic debris with no viable nuclei [24,25], thereby establishing the safety of IOCS in oncological surgery [36]. This is also supported by evidence from other studies [37] stating that upon passing through the IOCS system, 62% of tumor cells were destroyed, whereas the remaining 38% were morphologically altered.

Using flow cytometric studies, we then compared the number of CTCs present in the patient’s own blood with that in salvaged blood [38]. We found that salvaged blood contained a significantly lower number of CTCs than those present in the patient’s circulation [38]. Furthermore, we provided corroborative evidence that tumor cells passing through IOCS become nonviable and therefore cannot form new metastatic lesions [36]. We were able to demonstrate that CTCs lost the ability to develop into new metastatic lesions after passing through the IOCS apparatus, even without the use of leukocyte depletion filters (LDFs) [36]. LDFs are used to prevent leukocyte-mediated adverse reactions and have applications in both transplant surgery and treatment of hematological conditions [39]. LDFs are used for filtrating blood and have been proven to have the capability to remove tumor cells from the filtrate [38].

Currently, there is ample evidence in the literature for the clinical safety of salvaged blood used in oncological surgical procedures, including gastrointestinal [40], gynecological [41-43], hepatobiliary [40,44-46], and urological [47-54] surgical procedures (Multimedia Appendix 1 [55-64]). Although patients who received SBT required significantly lower amounts of allogeneic blood, their survival rates [44,49,51,52] and disease progression remained comparable with those who did not receive SBT [42,46,54,65]. Patients who received SBT had lower or similar rates of recurrence compared with the control cohort [47,49,51,53].

Despite the validity of the abovementioned literature, there still is hesitation to use IOCS in MSTS [34]. This can be addressed only by using a clinical study. Hence, we have designed this study to analyze the clinical use of IOCS in patients undergoing MSTS.

### Objectives

#### Primary Objectives

This study aims to investigate the following clinical outcomes: disease progression, in terms of tumor progression (increase in size of existing metastatic lesions with or without the appearance of new metastasis), and the overall survival (OS) in patients who receive *IOCS in combination with LDF* (IOCS-LDF)–processed blood during MSTS. Therefore, this study aims to refute the prevailing conception that cell salvage should be avoided in MSTS owing to concerns of tumor dissemination.

#### Secondary Objectives

The secondary objectives of the study are to investigate whether reinfusion of IOCS-LDF–processed blood can reduce ABT rates in patients undergoing MSTS. We will also compare the length of stay and overall complication rate of patients who receive salvaged blood and those who receive ABT or no blood transfusion (NBT).

### Hypothesis

The working hypotheses of this study are as follows:

1. Reinfusion of IOCS-LDF–processed blood of patients undergoing MSTS does not increase the risk of disease progression, in terms of tumor progression (increase in size of existing metastatic lesions with or without the appearance of new metastasis) and OS.
2. Patients receiving IOCS-LDF blood transfusion require less ABT.
3. Patients receiving IOCS-LDF blood transfusion will experience fewer overall complications and shorter length of stay than patients receiving ABT.

## Methods

### Recruitment

This study aims to recruit a minimum of 90 patients of whom 30 will receive SBT (with or with no allogeneic blood), 30 will undergo ABT, and the remaining 30 will have NBT. From our experience of treating patients with tumor, there are likely to be very few patients who receive only SBT. The majority of patients receiving SBT are likely to receive both SBT and ABT in various proportions. SBT and ABT form the 2 experimental arms, whereas patients with NBT form the control cohort. We will compare the number of patients receiving only SBT with those receiving ABT, if the sample sizes are sufficient.

Patients will be selected from specialist outpatient spine clinics or inpatient wards. These patients may be referred from an inpatient medical oncology team for management, especially in the setting of MSD with cord compression resulting in symptoms such as pain or neurology. A thorough examination of clinical history, physical examination, and review of imaging will be done, and appropriate patient management options will be discussed by the attending orthopedic surgeon. During the enrollment period, whenever a spine surgeon has obtained surgical consent for MSTS in patients with MSD, the principal investigator (NK) will be informed either by a telephone call or by text messaging.

The *research assistant* who is on-site at the National University Hospital during office hours or any member of the research team who is available will interview the patient. During this interview, the interviewer will confirm whether the inclusion criteria are fully met. The study will be explained to suitable subjects, including the advantages as well as possible intra- and postoperative risks of the reinfusion of salvaged blood. A copy of the *patient information sheet and consent form* will be provided to the patient, detailing the recruitment procedures, our objectives, hypotheses, and background information, and any queries will be addressed. Subsequently, written informed consent will be obtained from the patient.

Blood transfusion details will be collected from anesthetists immediately after the operation. Depending on the type of blood transfusion done during the surgery, that is, ABT only or SBT with or with no ABT or NBT, the patient will be categorized into the appropriate study cohort. All patients undergoing MSTS will receive tranexamic acid as an intravenous bolus before induction of anesthesia as per the department protocol, with subsequent top-up dosages every 4 hours during MSTS. If excessive blood loss is expected during surgery, such as in a separation surgery, continuous infusion of tranexamic acid is given to the patient.

Postoperatively, all subjects will be followed up individually by their operating surgeons, as per the standard of care at the National University Hospital. No additional patient follow-up sessions by the research team will be required. Clinical data and patient outcomes will be accessed via the computerized patient support system and EPIC system at monthly intervals. All available radiological data and clinical notes from the patients’ follow-up by their treating surgeons or physicians will be reviewed by the research team. This is to determine the postoperative outcomes such as survival and disease progression. The collected data will be analyzed postoperatively at 3, 6, 12, 24, 36, and 48 months. The data collection and follow-up of available records will continue over a period of 4 years postoperatively or until the patient’s demise, whichever occurs first. The collected outcomes of the 2 experimental groups (patients who received SBT with or without ABT) will be compared with those of the control group (patients who received NBT) as well as with the available historical data from the literature.

### Statistical Analysis

We have defined *disease progression* as the increase in the size of an existing metastatic lesion or the appearance of a new lesion in the lung, liver, or the spinal column, which can be visualized by using radiological imaging.

Demographic and clinical characteristics of patients will be summarized using mean (SD) values for continuous variables with approximately normal distribution, median (IQR) values for continuous variables with skewed distribution, and frequency (percentage) for categorical variables. A 1-way ANOVA will be used to compare the mean of a normally distributed variable across the 3 blood transfusion groups, whereas a Kruskal-Wallis rank test will be used for the comparison of medians. A Fisher exact test will be implemented for categorical variables accounting for potential small frequencies.

The association between individual characteristics and OS will be studied by using the Kaplan-Meier curve and a log-rank test. The crude hazard ratio and its 95% CI will be used to measure the association between individual characteristics and OS and will be calculated based on their original definitions. Multivariate Cox proportional hazard regression will then be used to adjust for statistically significant confounders for the relationship between the type of blood transfusion and OS. The proportional hazards assumption will be tested after the final model is obtained.

The association between the type of blood transfusion and tumor progression will be investigated by the competing risks analysis, taking death without tumor progression as the competing event. First, cumulative incidence curves of the 3 blood transfusion groups will be plotted nonparametrically, and then we will model the relation via a subdistribution hazard regression model. The measure of association will be quantified by the crude subdistribution hazard ratio; its 95% CI and *P* value will be analyzed in a univariate analysis. Subsequently, a multivariate analysis will be used to adjust for potential confounders. All statistical analyses will be done using Stata Special Edition 14.0 (StataCorp LP). The statistical tests will be assumed to be 2-sided, with the conventional 5% significance level.

#### Analysis of Primary Outcome Measures

We intend to compare the proportion of patients with MSTS requiring blood transfusion and the amount of ABT required. The primary analysis will be based on the intention-to-treat principle. A Fisher exact test will be used to compare the 2 arms. The exact 95% CI will also be calculated for the difference in the ABT rate between the 2 arms.

#### Analysis of Secondary Outcome Measures

The progression of disease will be assessed using the internationally accepted Response Evaluation Criteria in Solid Tumors (version 1.1) [66,67]. Disease progression is defined as at least a 20% increase in the sum of the diameters of measurable target lesions (eg, lymph nodes and bone metastases with soft tissue components), unequivocal progression of nontarget lesions (eg, malignant ascites or pleural effusions), or the appearance of 1 or more new metastatic lesions.

Computed tomography of chest or abdomen and pelvis will be used to assess metastases in the lymph nodes, lung, liver, or any abdominal organ, all of which can be visualized adequately. Magnetic resonance imaging of whole spine and nuclear medicine bone will also be performed because of their increased sensitivity in detecting early new spinal or skeletal metastases [68]. A lesion identified in a follow-up study in an anatomical location that is not present at baseline is considered a new lesion and will indicate disease progression.

The OS rate will be defined as the proportion of patients who survive until the end of the study period. Median survival times and 95% CI will be estimated using Kaplan-Meier curves for experimental and control groups. The median OS times will be compared using a log-rank test. The survival rate at 6 months after surgery will also be estimated using Kaplan-Meier curves.

### Ethical Considerations

The domain-specific review board of the National Healthcare Group, Singapore (reference numbers: 2014/00065 and 2022/00866), has granted ethical approval for this study. Written informed consent regarding participation in this research study and receiving SBT will be obtained from each patient before the patient is recruited for the study. Data have been anonymized and deidentified. No compensation was provided to patients for this study.

## Results

This study has been funded by the National Medical Research Council of Singapore in November 2016 and approved by domain-specific review board of the National Health Group in April 2016.

Data were collected from November 2016 to present (as of submission date of manuscript). Numbers recruited into study, as of submission of the manuscript, were 140. The status of data analysis and expected results are expected to be published in the years 2023-2024, when the information is available.

## Discussion

### Study Findings

This is the largest prospective clinical study on the use of IOCS in MSTS from a variety of primary malignancies. It will provide significant clinical evidence regarding the safety and applicability of IOCS in MSTS. The clinical safety of the use of IOCS has been established in oncological surgical procedures involving gastrointestinal [40], gynecological [41-43], hepatobiliary [40,44-46,55-57], and urological [47-54,58-62] specialties (Multimedia Appendix 1). However, the use of IOCS has neither been studied nor practiced regularly in metastatic musculoskeletal tumor surgeries (MMTSs). This may be because of the skepticism among surgeons about the safety of SBT in MMTSs, despite the presence of substantial supporting evidence in other surgical specialties in the field of oncology [40-54,63,64]. Amidst all the apprehension regarding IOCS, a retrospective comparative review has shown that SBT indeed reduces the need for postoperative ABT [69]. More recently, the use of SBT has been studied prospectively, demonstrating its safety for IOCS in MSTS [70]. Nonetheless, these studies did not have a comparative arm and had a small sample size.

This prospective clinical study is founded on substantiating evidence that salvaged blood is free from viable tumor cells, proven in our earlier methodical basic sciences approach [32,38]. We aim to study the OS, as well as tumor progression in MSTS patients through analysis of their various outcome measures. The results from this approach will help debunk the prevailing myth that IOCS contributes to disease progression either in the form of new metastasis or in the form of an increase in the size of the index lesion.

### Limitations

The limitation of this study is that not all patients undergoing MSTS will survive for the total follow-up period of 4 years, thereby theoretically leading to potential underreporting of disease progression. The sheer number of possible primary tumors in MSD also inevitably leads to heterogeneity, which can be overcome through propensity score–matching analysis.

### Broader Implications

Through the reporting of our analysis, this study will help reduce the use of ABT, reduce the burden on blood banks, and improve the overall blood management of patients with MSTS. This improvement in blood management will prevail even with the improvement of surgical techniques in the management of MSTS, that is, introduction of minimally invasive surgery techniques and the regular use of navigation. This is because the 2 techniques mentioned earlier will reduce blood loss during the steps of spinal instrumentation but are unlikely to have any effect on blood loss while performing decompression. Decompression and separation surgery presently still form a major component of MSTS and will continue to do so, resulting in significant bleeding that requires replenishment potentially in the form of SBT.

In this protocol, we have proposed a prospective observational nonrandomized study design as the ethical appropriateness of blinding or randomizing these patients is a key concern in our region and country. Blinding or randomization of patients with MSD could be deemed unethical, especially among patients who may not agree to receive SBT or ABT as this will limit the blood transfusion type applicable for them. This could be attributed to the current lack of clinical evidence that SBT does not lead to disease progression or shortened survival among patients undergoing MSTS who receive SBT. With this research proposal, we aim to highlight the safety profile of SBT, together with a design protocol applicable for use in patients with MSD. Future research, such as propensity-matched studies, can be done to further validate the outcomes from our current protocol.

### Conclusions

We surmise that the results of our proposed study design will pave the way for future randomized studies on the use of IOCS in MSTS and MMTSs, given that the granting bodies and their reviewers would be more open to considering funding for such studies.

## Appendix

Multimedia Appendix 1 Key papers evaluating the use of intraoperative cell salvage in various cancer surgeries.

## Abbreviations

ABT: allogeneic blood transfusion
CTC: circulating tumor cell
IOCS: intraoperative cell salvage
LDF: leukocyte depletion filter
MMTS: metastatic musculoskeletal tumor surgery
MSD: metastatic spine disease
MSTS: metastatic spine tumor surgery
NBT: no blood transfusion
OS: overall survival
SBT: salvaged blood transfusion

## Data Availability

All data generated or analyzed during this study will be included in published manuscripts and their supplementary information files.

## References

[1] Sciubba DM, Petteys RJ, Dekutoski MB, Fisher CG, Fehlings MG, Ondra SL, Rhines LD, Gokaslan ZL (2010). Diagnosis and management of metastatic spine disease. A review. J Neurosurg Spine.

[2] Witham TF, Khavkin YA, Gallia GL, Wolinsky J, Gokaslan ZL (2006). Surgery insight: current management of epidural spinal cord compression from metastatic spine disease. Nat Clin Pract Neurol.

[3] Choi D, Crockard A, Bunger C, Harms J, Kawahara N, Mazel C, Melcher R, Tomita K, Global Spine Tumor Study Group (2010). Review of metastatic spine tumour classification and indications for surgery: the consensus statement of the global spine tumour study group. Eur Spine J.

[4] Bilsky MH, Fraser JF (2006). Complication avoidance in vertebral column spine tumors. Neurosurg Clin N Am.

[5] Wang JC, Boland P, Mitra N, Yamada Y, Lis E, Stubblefield M, Bilsky MH (2004). Single-stage posterolateral transpedicular approach for resection of epidural metastatic spine tumors involving the vertebral body with circumferential reconstruction: results in 140 patients. Invited submission from the joint section meeting on disorders of the spine and peripheral nerves, March 2004. J Neurosurg Spine.

[6] Miller GV, Ramsden CW, Primrose JN (1991). Autologous transfusion: an alternative to transfusion with banked blood during surgery for cancer. Br J Surg.

[7] Valbonesi M, Bruni R, Lercari G, Florio G, Carlier P, Morelli F (1999). Autoapheresis and intraoperative blood salvage in oncologic surgery. Transfus Sci.

[8] Blajchman MA, Bordin JO (1995). The tumor growth-promoting effect of allogeneic blood transfusions. Immunol Invest.

[9] Blumberg N (1997). Allogeneic transfusion and infection: economic and clinical implications. Semin Hematol.

[10] Mynster T, Christensen IJ, Moesgaard F, Nielsen HJ (2000). Effects of the combination of blood transfusion and postoperative infectious complications on prognosis after surgery for colorectal cancer. Danish RANX05 colorectal cancer study group. Br J Surg.

[11] Zaw AS, Kantharajanna SB, Maharajan K, Tan B, Saparamadu AA, Kumar N (2017). Metastatic spine tumor surgery: does perioperative blood transfusion influence postoperative complications?. Transfusion.

[12] Kumar N, Chen Y, Nath C, Liu EHC (2012). What is the role of autologous blood transfusion in major spine surgery?. Am J Orthop (Belle Mead NJ).

[13] Menitove JE (2007). Transfusion related acute lung injury (TRALI): a review. Mo Med.

[14] Alshryda S, Sukeik M, Sarda P, Blenkinsopp J, Haddad FS, Mason JM (2014). Topical usage of tranexamic acid: comparative analysis in patients with bilateral total knee replacement. EC Orthopaedics.

[15] Kato S, Chikuda H, Ohya J, Oichi T, Matsui H, Fushimi K, Takeshita K, Tanaka S, Yasunaga H (2016). Risk of infectious complications associated with blood transfusion in elective spinal surgery—a propensity score matched analysis. Spine J.

[16] Zaw AS, Kantharajanna SB, Maharajan K, Tan B, Vellayappan B, Kumar N (2017). Perioperative blood transfusion: does it influence survival and cancer progression in metastatic spine tumor surgery?. Transfusion.

[17] Jacobs WB, Perrin RG (2001). Evaluation and treatment of spinal metastases: an overview. Neurosurg Focus.

[18] Spahn DR, Shander A, Hofmann A (2013). The chiasm: transfusion practice versus patient blood management. Best Pract Res Clin Anaesthesiol.

[19] Kang SB, Cho KJ, Moon KH, Jung J, Jung S (2011). Does low-dose aspirin increase blood loss after spinal fusion surgery?. Spine J.

[20] Korinth MC, Gilsbach JM, Weinzierl MR (2007). Low-dose aspirin before spinal surgery: results of a survey among neurosurgeons in Germany. Eur Spine J.

[21] Carless PA, Henry DA, Moxey AJ, O'Connell D, Brown T, Fergusson DA (2010). Cell salvage for minimising perioperative allogeneic blood transfusion. Cochrane Database Syst Rev.

[22] Liang J, Shen J, Chua S, Fan Y, Zhai J, Feng B, Cai S, Li Z, Xue X (2015). Does intraoperative cell salvage system effectively decrease the need for allogeneic transfusions in scoliotic patients undergoing posterior spinal fusion? A prospective randomized study. Eur Spine J.

[23] Williamson MDK, Taswell HF (1991). Intraoperative blood salvage: a review. Transfusion.

[24] Kumar N, Ahmed Q, Lee VKM, Zaw AS, Goy R, Wong HK (2016). Are we ready for the use of intraoperative salvaged blood in metastatic spine tumour surgery?. Eur Spine J.

[25] Catling S, Williams S, Freites O, Rees M, Davies C, Hopkins L (2008). Use of a leucocyte filter to remove tumour cells from intra-operative cell salvage blood. Anaesthesia.

[26] Waters JH, Yazer M, Chen YF, Kloke J (2012). Blood salvage and cancer surgery: a meta-analysis of available studies. Transfusion.

[27] Wells PS (2002). Safety and efficacy of methods for reducing perioperative allogeneic transfusion: a critical review of the literature. Am J Ther.

[28] McKenna SJ (1986). Autologous blood transfusions. Council on scientific affairs. JAMA.

[29] Yaw PB, Sentany M, Link WJ, Wahle WM, GGlover JL (1975). Tumor cells carried through autotransfusion. Contraindication to intraoperative blood recovery?. JAMA.

[30] Yamaguchi K, Takagi Y, Aoki S, Futamura M, Saji S (2000). Significant detection of circulating cancer cells in the blood by reverse transcriptase-polymerase chain reaction during colorectal cancer resection. Ann Surg.

[31] Allan AL, Keeney M (2010). Circulating tumor cell analysis: technical and statistical considerations for application to the clinic. J Oncol.

[32] Kumar N, Zaw A, Kantharajanna SB, Khoo BL, Lim CT, Thiery JP (2017). Metastatic efficiency of tumour cells can be impaired by intraoperative cell salvage process: truth or conjecture?. Transfus Med.

[33] Fidler IJ (1985). Macrophages and metastasis--a biological approach to cancer therapy. Cancer Res.

[34] Kumar N, Chen Y, Zaw AS, Nayak D, Ahmed Q, Soong R, Wong HK (2014). Use of intraoperative cell-salvage for autologous blood transfusions in metastatic spine tumour surgery: a systematic review. Lancet Oncol.

[35] Kumar N, Ahmed Q, Lee VK, Chen Y, Zaw AS, Goy R, Agrawal RV, Dhewar AN, Wong HK (2014). Can there be a place for intraoperative salvaged blood in spine tumor surgery?. Ann Surg Oncol.

[36] Kumar N, Zaw AS, Khoo BL, Nandi S, Lai Z, Singh G, Lim CT, Thiery JP (2016). Intraoperative cell salvage in metastatic spine tumour surgery reduces potential for reinfusion of viable cancer cells. Eur Spine J.

[37] Karczewski DM, Lema MJ, Glaves D (1994). The efficiency of an autotransfusion system for tumor cell removal from blood salvaged during cancer surgery. Anesth Analg.

[38] Kumar N, Lam R, Zaw AS, Malhotra R, Tan J, Tan G, Setiobudi T (2014). Flow cytometric evaluation of the safety of intraoperative salvaged blood filtered with leucocyte depletion filter in spine tumour surgery. Ann Surg Oncol.

[39] Singh S, Kumar A (2009). Leukocyte depletion for safe blood transfusion. Biotechnol J.

[40] Bower MR, Ellis SF, Scoggins CR, McMasters KM, Martin RCG (2011). Phase II comparison study of intraoperative autotransfusion for major oncologic procedures. Ann Surg Oncol.

[41] Beck-Schimmer B, Romero B, Booy C, Joch H, Hallers U, Pasch T, Spahn DR (2004). Release of inflammatory mediators in irradiated cell salvage blood and their biological consequences in human beings following transfusion. Eur J Anaesthesiol.

[42] Connor JP, Morris PC, Alagoz T, Anderson B, Bottles K, Buller RE (1995). Intraoperative autologous blood collection and autotransfusion in the surgical management of early cancers of the uterine cervix. Obstet Gynecol.

[43] Mirhashemi R, Averette HE, Deepika K, Estape R, Angioli R, Martin J, Rodriguez M, Penalver MA (1999). The impact of intraoperative autologous blood transfusion during type III radical hysterectomy for early-stage cervical cancer. Am J Obstet Gynecol.

[44] Fujimoto J, Okamoto E, Yamanaka N, Oriyama T, Furukawa K, Kawamura E, Tanaka T (1991). [Autotransfusion in hepatectomy for hepatocellular carcinoma]. Nihon Geka Gakkai Zasshi.

[45] Hirano T, Yamanaka J, Iimuro Y, Fujimoto J (2005). Long-term safety of autotransfusion during hepatectomy for hepatocellular carcinoma. Surg Today.

[46] Kim JM, Kim GS, Joh J, Suh K, Park JB, Ko JS, Kwon CHD, Yi N, Gwak MS, Lee K, Kim SJ, Lee S (2013). Long-term results for living donor liver transplant recipients with hepatocellular carcinoma using intraoperative blood salvage with leukocyte depletion filter. Transpl Int.

[47] Davis M, Sofer M, Gomez-Marin O, Bruck D, Soloway MS (2003). The use of cell salvage during radical retropubic prostatectomy: does it influence cancer recurrence?. BJU Int.

[48] Ford BS, Sharma S, Rezaishiraz H, Huben RS, Mohler JL (2008). Effect of perioperative blood transfusion on prostate cancer recurrence. Urol Oncol.

[49] Nieder AM, Carmack AJ, Sved PD, Kim SS, Manoharan M, Soloway MS (2005). Intraoperative cell salvage during radical prostatectomy is not associated with greater biochemical recurrence rate. Urology.

[50] Nieder AM, Manoharan M, Yang Y, Soloway MS (2007). Intraoperative cell salvage during radical cystectomy does not affect long-term survival. Urology.

[51] Gorin MA, Eldefrawy A, Manoharan M, Soloway MS (2012). Oncologic outcomes following radical prostatectomy with intraoperative cell salvage. World J Urol.

[52] Klimberg I, Sirois R, Wajsman Z, Baker J (1986). Intraoperative autotransfusion in urologic oncology. Arch Surg.

[53] MacIvor D, Nelson J, Triulzi D (2009). Impact of intraoperative red blood cell salvage on transfusion requirements and outcomes in radical prostatectomy. Transfusion.

[54] Park KI, Kojima O, Tomoyoshi T (1997). Intra-operative autotransfusion in radical cystectomy. Br J Urol.

[55] Foltys D, Zimmermann T, Heise M, Kaths M, Lautem A, Wisser G, Weiler N, Hoppe-Lotichius M, Hansen T, Otto G (2011). Liver transplantation for hepatocellular carcinoma--is there a risk of recurrence caused by intraoperative blood salvage autotransfusion?. Eur Surg Res.

[56] Han S, Kim G, Ko JS, Sinn DH, Yang JD, Joh J, Lee S, Gwak MS (2016). Safety of the use of blood salvage and autotransfusion during liver transplantation for hepatocellular carcinoma. Ann Surg.

[57] Araujo RL, Pantanali CA, Haddad L, Rocha Filho JA, D'Albuquerque LAC, Andraus W (2016). Does autologous blood transfusion during liver transplantation for hepatocellular carcinoma increase risk of recurrence?. World J Gastrointest Surg.

[58] Gray CL, Amling CL, Polston GR, Powell CR, Kane CJ (2001). Intraoperative cell salvage in radical retropubic prostatectomy. Urology.

[59] Stoffel JT, Topjian L, Libertino JA (2005). Analysis of peripheral blood for prostate cells after autologous transfusion given during radical prostatectomy. BJU Int.

[60] Ubee S, Kumar M, Athmanathan N, Singh G, Vesey S (2011). Intraoperative red blood cell salvage and autologous transfusion during open radical retropubic prostatectomy: a cost-benefit analysis. Ann R Coll Surg Engl.

[61] Raval JS, Nelson JB, Woldemichael E, Triulzi DJ (2012). Intraoperative cell salvage in radical prostatectomy does not appear to increase long-term biochemical recurrence, metastases, or mortality. Transfusion.

[62] Kinnear N, Heijkoop B, Hua L, Hennessey DB, Spernat D (2018). The impact of intra-operative cell salvage during open radical prostatectomy. Transl Androl Urol.

[63] Perseghin P, Viganò M, Rocco G, Della Pona C, Buscemi A, Rizzi A (1997). Effectiveness of leukocyte filters in reducing tumor cell contamination after intraoperative blood salvage in lung cancer patients. Vox Sang.

[64] Chalfin HJ, Frank SM, Feng Z, Trock BJ, Drake CG, Partin AW, Humphreys E, Ness PM, Jeong BC, Lee SB, Han M (2014). Allogeneic versus autologous blood transfusion and survival after radical prostatectomy. Transfusion.

[65] Muscari F, Suc B, Vigouroux D, Duffas J, Migueres I, Mathieu A, Lavayssiere L, Rostaing L, Fourtanier G (2005). Blood salvage autotransfusion during transplantation for hepatocarcinoma: does it increase the risk of neoplastic recurrence?. Transpl Int.

[66] Eisenhauer EA, Therasse P, Bogaerts J, Schwartz LH, Sargent D, Ford R, Dancey J, Arbuck S, Gwyther S, Mooney M, Rubinstein L, Shankar L, Dodd L, Kaplan R, Lacombe D, Verweij J (2009). New response evaluation criteria in solid tumours: revised RECIST guideline (version 1.1). Eur J Cancer.

[67] van Persijn van Meerten EL, Gelderblom H, Bloem JL (2010). RECIST revised: implications for the radiologist. A review article on the modified RECIST guideline. Eur Radiol.

[68] Lecouvet F, Talbot JN, Messiou C, Bourguet P, Liu Y, de Souza NM, EORTC Imaging Group (2014). Monitoring the response of bone metastases to treatment with magnetic resonance imaging and nuclear medicine techniques: a review and position statement by the European organisation for research and treatment of cancer imaging group. Eur J Cancer.

[69] Elmalky M, Yasin N, Rodrigues-Pinto R, Stephenson J, Carroll C, Smurthwaite G, Verma R, Mohammad S, Siddique I (2017). The safety, efficacy, and cost-effectiveness of intraoperative cell salvage in metastatic spine tumor surgery. Spine J.

[70] Zong Y, Xu C, Gong Y, Zhang X, Zeng H, Liu C, Zhang B, Xue L, Guo X, Wei F, Li Y (2022). Effectiveness of intraoperative cell salvage combined with a modified leucocyte depletion filter in metastatic spine tumour surgery. BMC Anesthesiol.

