# Supplementary material for: Evaluation of the Feasibility of Transfusing Leukocyte Depletion Filter–Processed Intraoperative Cell Salvage Blood in Metastatic Spine Tumor Surgery: Protocol for a Non–Randomized Study
Source: JMIR Res Protoc. 2025 Jan 17;14:e54609. doi: 10.2196/54609 (PMC11786134; doi:10.2196/54609)
Supplement: Multimedia Appendix 1 [file resprot_v14i1e54609_app1.docx]

| Type of surgery | Reference | Country | Type of study | Number of patients | Level of evidence | Follow-up period | Outcomes | Tumor recurrence | OS^a^ |
| --- | --- | --- | --- | --- | --- | --- | --- | --- | --- |
| **Gynecological** | | | | | | | | | |
|  | Connor et al, 1995 [42] | United States | Prospective cohort | - IOCS^b^=31 - Non-IOCS=40 | 3 | Mean 24 months | IOCS decreases the need for intra- and postoperative ABT^c^. IOCS does not appear to reinfuse tumor cells in patients undergoing radical hysterectomy for cervical cancer | —^d^ | — |
|  | Mirhashemi et al, 1999 [43] | United States | Retrospective cohort | - IOCS=50 - Non-IOCS=106 | 3 | Mean 22 months | IOCS group (12%) received significantly less ABT as compared with the non-IOCS group (30%; *P*=.02). No significant differences were studied between IOCS and non-IOCS groups across all 11 postoperative complications | — | — |
|  | Catling et al, 2008 [25] | United Kingdom | Prospective observational cohort | - IOCS=50 | 2 | N/A^e^ | No viable nucleated malignant cells were detectable in any of the final filtered samples of cell-salvaged blood | — | — |
| **Hepatobiliary** | | | | | | | | | |
|  | Fujimoto et al, 1991 [44] | Japan | Prospective cohort | - IOCS=54 - ABT=50 | 3 | Until death or predefined study end date (December 1991) | Mean volume of allogeneic blood transfused was significantly less in the IOCS group (814 mL, SD 397 mL) vs the ABT group (3466 mL, SD 1811 mL); *P*<.05 | No significant differences between IOCS and ABT groups in terms of cumulative recurrence rates (62.8% vs 67.3%) | No significant differences between IOCS and ABT groups in terms of cumulative survival rates (61.9% vs 52.8%) |
|  | Muscari et al, 2005 [55] | France | Prospective cohort | - IOCS=31 - Non-IOCS=16 | 3 | Median (months): IOCS=48 and non-IOCS=15 | No significant difference in tumor recurrence rates between IOCS and non-IOCS groups | No significant difference in tumor recurrence rates between IOCS (6.4%) and non-IOCS (6.3%) groups | — |
|  | Foltys et al, 2011 [59] | Germany | Prospective cohort | - IOCS=40 - Non-IOCS=96 | 3 | Median (days): IOCS=1146 and non-IOCS=877 | No significant difference in tumor recurrence rates between IOCS and non-IOCS groups | No significant difference in tumor recurrence rates between IOCS (5/40, 13% patients) and non-IOCS (18/96, 19% patients) groups (*P*=.29) | — |
|  | Kim et al, 2013 [46] | Korea | Retrospective cohort | - IOCS=121 - Non-IOCS=109 | 3 | Median (months): IOCS=53 and non-IOCS=33 | Average volume of allogeneic blood transfused was significantly less in IOCS group (3.7 units) vs non-IOCS group (9.9 units); *P*<.01 | — | No significant difference in RFS^f^ rates between IOCS (83.3%) and non-IOCS (77.4%) groups (*P*=.31) |
|  | Han et al, 2016 [60] | Korea | Retrospective cohort | - IOCS=283 - Non-IOCS=114 | 3 | 5 years | No significant impact of auto transfusion on posttransplant HCC^g^ recurrence | Cumulative posttransplantation HCC recurrence rate at 1, 2, and 5 years were 10.4% (5.3%-17.6%), 19.1% (11.6%-28.0%), and 24.1% (15.2%-34.0%), respectively, for nonauto transfusion group and 10.8% (7.2%-15.4%), 14.9% (10.5%-20.0%), and 20.3% (14.9%-26.4%) for auto transfusion group, respectively | — |
|  | Araujo et al, 2016 [61] | Brazil | Retrospective cohort | - IOCS=122 - Non-IOCS=36 | 3 | 5 years | No differences in OS (*P*=.51) or RFS (*P*=.95) were detected between the IOCS and non-IOCS groups | — | The OS and RFS at 5 years were 59.7% and 83.3%, respectively |
| **Gastrointestinal** | | | | | | | | | |
|  | Bower et al, 2011 [40] | United States | Prospective cohort | - IOCS=32 - Non-IOCS=60 | 3 | Median 18 months | At median follow-up, no significant difference between the IOCS group and non-IOCS group was observed in terms of overall recurrence rates, pancreatic cancer recurrence rates, and locoregional recurrence rates. Distant recurrence rates were lower in IOCS vs non-IOCS group, although not statistically significant | IOCS group vs non-IOCS group:   - Overall recurrence rates (28% vs 43%; *P*=.9) - Pancreatic cancer recurrence rates (33% vs 24%; *P*=.7) - Locoregional recurrence rates (22% vs 17%; *P*=.58) - Distant recurrence rates were lower in IOCS vs non-IOCS group, although not statistically significant (16% vs 25%; *P*=.43) | — |
| **Urology** | | | | | | | | | |
|  | Park et al, 1999 [54] | Japan | Prospective cohort | - IOCS=6 - IOCS+PAD^h^=4 | 3 | Mean 47 months | IOCS seems feasible in reducing or avoiding ABT in radical cystectomy. IOCS+PAD will abolish the need for ABT in radical cystectomy | — | — |
|  | Gray et al, 2001 [62] | United States | Prospective cohort | - IOCS-LDF^i^=62 - PAD=101 | 3 | Mean (months): IOCS-LDF group=7 and PAD group=43 | PAD and IOCS-LDF groups (*P*=.41). ABT requirements were significantly lower in the IOCS-LDF group (3%) vs the PAD (14%) group (*P*=.04) | — | — |
|  | Davis et al, 2003 [47] | United States | Retrospective cohort | - IOCS=87 - PAD=264 - No transfusion=57 | 3 | Mean 40.2 months | No significant difference in biochemical recurrence rates among the IOCS, PAD, and no transfusion groups. The IOCS group was less likely to have recurrence compared with the PAD group and no transfusion group | No significant difference in biochemical recurrence rates among the IOCS (15%), PAD (16%), and no transfusion (19%) groups (*P*=.78). The IOCS group was less likely to have recurrence compared with the PAD group (OR^j^ 0.81, 95% CI 0.33-2.00) and no transfusion group (OR 0.66, 95% CI 0.21-2.08) | — |
|  | Neider et al, 2005 [49] | United States | Retrospective cohort | - IOCS=265 - Non-IOCS=773 | 3 | Median (months): IOCS=40.3 and non-IOCS=44.4 | No significant difference in biochemical recurrence rates at 5 years and the RFS between the IOCS group and non-IOCS group | No significant difference in biochemical recurrence rates at 5 years between the IOCS group (15%) and non-IOCS group (18%; *P*=.76) | No significant difference in RFS between the IOCS group (27.9 months, SD 30.3) and non-IOCS group (32.1 months, SD 29.5; *P*=.49) |
|  | Stoffel et al, 2005 [63] | United States | Prospective cohort | - IOCS=48 - Non-IOCS=64 | 3 | Mean (months): IOCS=46 and non-IOCS=43 | Cox regression showed that advanced pathological stage and Gleason score, but not IOCS, were independent predictors of biochemical failure | IOCS: adjusted hazard ratio for biochemical recurrence=0.766 (*P*=.54) | — |
|  | Nieder et al, 2007 [50] | United States | Retrospective cohort | - IOCS=65 - Non-IOCS=313 | 3 | Median (months): IOCS=19.1 and non-IOCS=20.7 | No significant difference in the disease-specific survival rate for IOCS and non-IOCS groups. IOCS is a safe blood management method for patients undergoing radical cystectomy | — | No significant difference in the disease-specific survival rate for IOCS and non-IOCS groups, 72.2% and 73.0%, respectively (*P*=.9) |
|  | Ford et al, 2008 [48] | United States | Prospective cohort | - IOCS=252 - ABT=117 - No transfusion=242 | 3 | Mean 44 months | No significant difference in biochemical failure rates among IOCS (10%), ABT (16%), and no transfusion groups (14%; *P*=.42). | — | No significant difference in biochemical progression-free survival rates at 5 years among no transfusion (86%), IOCS (90%), and ABT groups (84%; *P*=.42) |
|  | Ubee et al, 2011 [64] | United Kingdom | Prospective cohort | - IOCS=25 - Non-IOCS=25 | 3 | 3 months | Overall, 20% of patients in the IOCS group received a total of 16 units of homologous blood, whereas 72% in the non-IOCS group received a total of 69 units. No evidence was found that IOCS increased the risk of early biochemical relapse or tumor dissemination | Overall, 16% in the non-IOCS group had biochemical recurrence vs 4% in the IOCS group | — |
|  | Gorin et al, 2012 [51] | United States | Retrospective cohort | - IOCS=395 - Non-IOCS=1467 | 3 | Median (months): IOCS=47 and non-IOCS=48 | Overall, 0.6% of patients in the IOCS group required ABT vs 3% to 20% in the non-IOCS group. IOCS use was not associated with an increased risk of BCR^k^ and decrease in OS | — | The IOCS group had 5-year and 8-year BCR-free survival rates of 82.4% and 73.4%, respectively, whereas the non-IOCS group had survival rates of 83.7% and 76.6%, respectively (*P*=.32). |
|  | Raval et al, 2012 [65] | United States | Retrospective cohort | - IOCS=63 - PAD=53 | 3 | Mean (days): IOCS=1857 and PAD=1977 | Patients with evidence of metastatic disease were significantly fewer in the IOCS group (0%) than PAD group (12.5%; *P*=.03). | Patients with biochemical recurrence were significantly fewer in the IOCS group (9.5%) than PAD group (34.4%; *P*=.02). | — |
|  | Kinnear et al, 2018 [66] | Australia | Retrospective cohort | - IOCS=29 - Non-IOCS=30 | — | IOCS=989 days and non-IOCS=945 days | Complications: IOCS=5; non-IOCS=10 (*P*=.16). IOCS reduced transfusion-related costs, without affecting allogeneic transfusion rates, tumor recurrence, or complication rates | Tumor recurrence: IOCS=3; non-IOCS=6 (*P*=.30) | — |
| **Lung** | | | | | | | | | |
|  | Perseghin et al, 1997 [67] | Italy | Prospective observational cohort | - IOCS=16 | 3 | N/A | Tumor cells were detected in 9 out of 16 (56%) samples post-IOCS. In the postleukocyte depletion filter samples, only cell debris or damaged mononuclear cells were detectable; no malignant tumor cells were detected | — | — |
| **Prostate** | | | | | | | | | |
|  | Chalfin et al, 2014 [68] | United States | Retrospective cohort | - IOCS only=5124 - ABT with or without IOCS=258 - No blood transfusion =2061 | 3 | Median 6 years | In the multivariate models, neither autologous nor ABT was independently associated with biochemical recurrence–free survival, cancer-specific survival, or OS | — | — |

^a^OS: overall survival.

^b^IOCS: intraoperative cell salvage.

^c^ABT: allogeneic blood transfusion.

^d^: not mentioned in the study

^e^N/A: not applicable.

^f^RFS: recurrence-free survival.

^g^HCC: hepatocellular carcinoma

^h^PAD: preoperative autologous donation.

^i^IOCS-LDF: intraoperative cell salvage in combination with leukocyte depletion filter.

^j^OR: odds ratio.

^k^BCR: biochemical recurrence

1. Catling S, Williams S, Freites O, et al. Use of a leucocyte filter to remove tumour cells from intra‐operative cell salvage blood. Anaesthesia 2008;63(12):1332-38.
   PMID: 19032302
2. Bower MR, Ellis SF, Scoggins CR, et al. Phase II comparison study of intraoperative autotransfusion for major oncologic procedures. Annals of surgical oncology 2011;18(1):166-73. PMID: 21222043
3. Connor JP, Morris PC, Alagoz T, et al. Intraoperative autologous blood collection and autotransfusion in the surgical management of early cancers of the uterine cervix. Obstetrics & Gynecology 1995;86(3):373-78. PMID: 7651645
4. Mirhashemi R, Averette HE, Deepika K, et al. The impact of intraoperative autologous blood transfusion during type III radical hysterectomy for early-stage cervical cancer. American Journal of Obstetrics & Gynecology 1999;181(6):1310-16. PMID: 10601905
5. Fujimoto J, Okamoto E, Yamanaka N, et al. Autotransfusion in hepatectomy for hepatocellular carcinoma. Nihon Geka Gakkai zasshi 1991;92(7):825-30.
   PMID: 1653401
6. Hirano T, Yamanaka J, Iimuro Y, et al. Long-term safety of autotransfusion during hepatectomy for hepatocellular carcinoma. Surgery today 2005;35(12):1042-46.
   PMID: 16341484
7. Kim JM, Kim GS, Joh JW, et al. Long‐term results for living donor liver transplant recipients with hepatocellular carcinoma using intraoperative blood salvage with leukocyte depletion filter. Transplant International 2013;26(1):84-89.
   PMID: 23194351
8. Davis M, Sofer M, Gomez‐Marin O, et al. The use of cell salvage during radical retropubic prostatectomy: does it influence cancer recurrence? BJU international 2003;91(6):474-76. PMID: 12656896
9. Ford BS, Sharma S, Rezaishiraz H, Huben RS, Mohler JL, editors. Effect of perioperative blood transfusion on prostate cancer recurrence. Urologic Oncology: Seminars and Original Investigations; 2008;26:364-367
   PMID: 18367097
10. Nieder AM, Carmack AJ, Sved PD, et al. Intraoperative cell salvage during radical prostatectomy is not associated with greater biochemical recurrence rate. Urology 2005;65(4):730-34. PMID: 15833517
11. Nieder AM, Manoharan M, Yang Y, et al. Intraoperative cell salvage during radical cystectomy does not affect long-term survival. Urology 2007;69(5):881-84.
    PMID: 17482926
12. Gorin MA, Eldefrawy A, Manoharan M, et al. Oncologic outcomes following radical prostatectomy with intraoperative cell salvage. World journal of urology 2012;30(3):379-83. PMID: 21847657
13. Park K, Kojima O, Tomoyoshi T. Intra‐operative autotransfusion in radical cystectomy. BJU International 1997;79(5):717-21. PMID: 9158508
14. Muscari F, Suc B, Vigouroux D, et al. Blood salvage autotransfusion during transplantation for hepatocarcinoma: does it increase the risk of neoplastic recurrence? Transplant international 2005;18(11):1236-39. PMID: 16221153
15. Foltys D, Zimmermann T, Heise M, et al. Liver transplantation for hepatocellular carcinoma–is there a risk of recurrence caused by intraoperative blood salvage autotransfusion? European Surgical Research 2011;47(3):182-87.
    PMID: 21986299
16. Han S, Kim G, Ko JS, et al. Safety of the use of blood salvage and autotransfusion during liver transplantation for hepatocellular carcinoma. Annals of surgery 2016;264(2):339-43. PMID: 26501715
17. Araujo RL, Pantanali CA, Haddad L, et al. Does autologous blood transfusion during liver transplantation for hepatocellular carcinoma increase risk of recurrence? World journal of gastrointestinal surgery 2016;8(2):161. PMID: 26981190
18. Gray CL, Amling CL, Polston GR, et al. Intraoperative cell salvage in radical retropubic prostatectomy. Urology 2001;58(5):740-45. PMID: 11711352
19. Stoffel JT, Topjian L, Libertino JA. Analysis of peripheral blood for prostate cells after autologous transfusion given during radical prostatectomy. BJU international 2005;96(3):313-15. PMID: 16042720
20. Ubee S, Kumar M, Athmanathan N, et al. Intraoperative red blood cell salvage and autologous transfusion during open radical retropubic prostatectomy: a cost–benefit analysis. The Annals of The Royal College of Surgeons of England 2011;93(2):157-61. PMID: 22041147
21. Raval JS, Nelson JB, Woldemichael E, et al. Intraoperative cell salvage in radical prostatectomy does not appear to increase long‐term biochemical recurrence, metastases, or mortality. Transfusion 2012;52(12):2590-93. PMID: 22612661
22. Kinnear N, Heijkoop B, Hua L, et al. The impact of intra-operative cell salvage during open radical prostatectomy. Translational Andrology and Urology 2018:S179-S87. PMID: 29928615
23. Perseghin P, Vigano M, Rocco G, et al. Effectiveness of leukocyte filters in reducing tumor cell contamination after intraoperative blood salvage in lung cancer patients. Vox sanguinis 1997;72(4):221-24. PMID: 9228711
24. Chalfin HJ, Frank SM, Feng Z, et al. Allogeneic versus autologous blood transfusion and survival after radical prostatectomy (CME). Transfusion 2014;54(9):2168-74. PMID: 24601996
